# A six‐plex droplet digital RT‐PCR assay for seasonal influenza virus typing, subtyping, and lineage determination

**DOI:** 10.1111/irv.12769

**Published:** 2020-06-10

**Authors:** Nathaniel K. C. Leong, Daniel K. W. Chu, Julie T. S. Chu, Yat H. Tam, Dennis K. M. Ip, Benjamin J. Cowling, Leo L. M. Poon

**Affiliations:** ^1^ School of Public Health LKS Faculty of Medicine The University of Hong Kong Hong Kong China

**Keywords:** digital RT‐PCR, influenza, RT‐PCR

## Abstract

**Background:**

There are two influenza A subtypes (H1 and H3) and two influenza B lineages (Victoria and Yamagata) that currently co‐circulate in humans. In this study, we report the development of a six‐plex droplet digital RT‐PCR (ddRT‐PCR) assay that can detect HA and M segments of influenza A (H1, H3, and M) and influenza B (Yamagata HA, Victoria HA, and M) viruses in a single reaction mixture. It can simultaneously detect six different nucleic acid targets in a ddRT‐PCR platform.

**Methods:**

The six‐plex ddRT‐PCR used in this study is an amplitude‐based multiplex assay. The analytical performance of the assay was evaluated. Correlation with standard qRT‐PCR methodology was assessed using 55 clinical samples.

**Results:**

The assay has a wide dynamic range, and it has good reproducibility within and between runs. The limit of quantification of each target in this assay ranged from 15 copies/reaction for influenza B Victoria M gene to 45 copies/reaction for influenza B Yamagata M gene. In addition, this assay can accurately quantify each of these targets in samples containing viral RNAs from two different viruses that were mixed in a highly skewed ratio. Typing, subtyping, and lineage differentiation data of 55 tested clinical respiratory specimens were found to be identical to those deduced from standard monoplex qRT‐PCR assays.

**Conclusions:**

The six‐plex ddRT‐PCR test was demonstrated to be highly suitable for detecting dual influenza infection cases. This assay is expected to be a useful diagnostic tool for clinical and research use.

## 1 INTRODUCTION

Influenza viruses belong to the *Orthomyxoviridae* family, and both human influenza A and B viruses can cause seasonal epidemics.^1^ Seasonal influenza is a highly contagious respiratory disease. About 5%‐10% of adults and 20%‐30% of children are infected by seasonal influenza viruses each year. Currently, there are two subtypes of influenza A (H1N1: H1pdm09 and H3N2: H3) and two lineages of influenza B virus (Yamagata: Yam and Victoria: Vic) that co‐circulate in humans. The circulating human influenza A H1N1 subtype emerged from pigs in the 2009 pandemic, and it replaced the classical seasonal H1N1 virus thereafter. Viruses of the four subtypes/lineages circulate widely in humans and 0.5%‐3.0% of influenza patients are dually infected.^2^, ^3^ Such dual infection cases are reported, but not necessarily confined to, children, young adults, pregnant women, and immunocompromised patients.^2^, ^4^, ^5^, ^6^

Quantitative RT‐PCR (qRT‐PCR) is a sensitive and specific nucleic acid test for detecting influenza virus.^7^ Viral loads determined by qRT‐PCR assays are suggested to be useful markers for assessing disease severity and for predicting clinical outcomes.^8^, ^9^, ^10^ However, quantification of nucleic acid targets in standard qRT‐PCR assays heavily relies on the quality of external standards and the relative signal‐to‐noise ratio.^11^, ^12^ Thus, the performance, reproducibility, and amplification efficiency of a standard qRT‐PCR‐based assay can vary greatly between different laboratories. The recent development of droplet digital PCR (ddPCR) provides an alternative solution to overcome this potential hurdle. The ddRT‐PCR assay uses microfluidics and emulsion chemistries to generate about 20 000 partitions or droplets per reaction.^13^ With each of these emulsified droplets containing approximately ≤ 1 copy of studied targets, quantification of copy numbers can be reliably calculated using Poisson statistics.^14^ Thus, the ddPCR approach does not require the use of a standard curve for quantification.

Multiplex assays have several advantages over standard monoplex assays, in terms of reducing reagent cost, sample consumption, hands‐on processing time, accumulated pipetting inaccuracy etc.^15^, ^16^ There are real‐time multiplex qRT‐PCR assays for typing and subtyping seasonal influenza viruses. Some can even detect influenza B virus and determine influenza B viral lineage,^17^, ^18^ but none of these assays allows simultaneous typing and subtyping/lineage differentiation of all 4 seasonal influenza viruses in a single qRT‐PCR reaction. Further, ddRT‐PCR does not require a standard curve for quantification and it is less susceptible to PCR inhibitors.^19^ Here, we report an efficient six‐plex ddRT‐PCR that can enhance influenza surveillance and epidemiologic studies thereby informing immunization policies, control strategies, and outbreak responses. The assay allows influenza virus typing and subtype/lineage determination in a single reaction.

## 2 METHODS AND MATERIALS

### 2.1 Primers and probes

Primers and probes targeting human influenza viruses (A: H1pdm09 or H3; B: Vic or Yam) were designed based on sequences available in the public domain (Influenza Research Database; period 2009‐2019) (Table 1). Studied influenza B virus HA sequences were classified into Vic or Yam lineage using neighbor‐joining phylogenetic analyses in MEGA X.^20^ Primer‐probe sets targeting the conserved region of influenza types A or B M genes were used for typing, whereas primer‐probe sets targeting the corresponding HA gene of seasonal influenza viruses (ie, H1pdm09, H3, Vic, or Yam) were used for subtyping or lineage differentiation. This established ddRT‐PCR consisted of five primer‐probe sets: influenza A M gene (Set 1), seasonal influenza A H3 HA gene (Set 2), influenza A H1pdm09 HA gene (Set 3), influenza B M gene (Set 4), and influenza B HA gene (Set 5). Set 5 contained two primers that can bind to both Vic and Yam HA sequences and two specific probes that can hybridize to Vic or Yam HA sequences. The specificities of these primer‐probe sets were studied using an in‐house R script. A sequence from the database was considered as a closely or perfectly matched sequence to a primer if it fulfilled all of the following criteria: (a) the last five bases at the 3′‐end of a primer should be a perfect match to its target; (b) the last ten bases at the 3′‐end of a primer should not have more than one mismatch to its target; and (c) the maximum number of mismatches between a primer and its target should not be more than two. A sequence from the database was considered as closely or perfectly matched to the probe sequence if not more than one mismatch was found. All primers and probes were synthesized commercially (Integrated DNA Technologies). All the probes were labeled with a 5′‐ fluorophore (FAM or HEX), a 3′‐ Iowa Black FQ quencher and an internal ZEN quencher.

**TABLE 1:** Primer/probe set design

Primers & probes	Sequence (5′ → 3′)	Database downloaded date^c^	Total sequences^d^	No. of matched sequences (%)
Set 1: Influenza A M gene				
FluA‐M‐F	CTTCTAACCGAGGTCGAAACGTA	18/9/2019	7983	99.97
FluA‐M‐R	AGGGCATTYTGGACAAAKCGTCTA	18/9/2019	7983	99.72
FluA‐M‐FAM‐F^a^	TCAGGCCCCCTCAAAGCCGAG	18/9/2019	7983	100.00
Set 2: Seasonal influenza A H3 HA gene				
H3‐HA‐F	GCGCAATMGCGGGTTTCATAG	16/10/2019	8034	99.58
H3‐HA‐R	CCTCTYCCCTCAGAATTTTGATGCCTG	16/10/2019	8034	98.98
H3‐HA‐HEX‐F^b^	TTGGGAGGGAATGGTGGATGGTTGGTACGG	16/10/2019	8034	97.55
Set 3: Influenza A pandemic 2009 H1 HA gene				
H1pdm09‐HA‐F	GTGCTATAAACACCAGCCTCCCA	16/10/2019	5043	98.45
H1pdm09‐HA‐R	AGAYGGGACATTCCTCAATCCTG	16/10/2019	5043	99.27
H1pdm09‐HA‐HEX‐F^b^	ATGTAAAAAGCACAAAATTGAGACTGGCCA	16/10/2019	5043	99.86
Set 4: Influenza B M gene				
FluB‐M‐F	GAGACACAATTGCCTACYTGCTT	22/9/2019	3191	99.94
FluB‐M‐R	CAAATTCTTTCCCACCRAACCAAC	22/9/2019	3191	99.84
FluB‐M‐FAM‐F^a^	AGAAGATGGAGAAGGCAAAGCAGAACTAGC	22/9/2019	3191	99.87
Set 5: Influenza B HA gene				
FluB‐HA‐F	AGGRGAAGACCAAATTACYGTTTG	16/10/2019	4404	98.89
FluB‐HA‐R	CRTTRGCAGATGAGGTGAACTT	16/10/2019	4404	99.07
FluB‐Yam‐HA‐HEX‐F^b^	ATRACAAARCCCAAATGAAGARCCTCTA	16/10/2019	2696	99.81
FluB‐Vic‐HA‐FAM‐F^a^	YARCGAGRYCCAAATGGHAARSCTCTATG	16/10/2019	1708	97.01

^a^ Probe format: PrimeTime^®^ 5′ 6‐FAM/ZEN/3′ IB^®^FQ.

^b^ Probe format: PrimeTime^®^ 5′ HEX/ZEN/3′ IB^®^FQ.

^c^ Coverage years: 2009‐2019.

^d^ Complete human influenza viral genome only.

### 2.2 Virus stock and viral nucleic acid extraction

Four laboratory strains were selected for assay development and evaluation. These were A/California/7/2009 (H1N1), A/Hong Kong/1/1968 (H3N2), B/Hong Kong/407373/2011 (Victoria), and B/Taiwan/N1902/2004 (Yamagata). These viruses were cultured in MDCK cells. Viral RNA was extracted from supernatants of infected cultures using QIAamp viral RNA mini kit (Qiagen) according to the manufacturer's instruction. All RNA samples were stored at −80°C before use.

### 2.3 Six‐plex ddRT‐PCR

For developing a six‐plex ddRT‐PCR, reactions were prepared using a one‐step ddRT‐PCR Advanced Kit for Probes (Bio‐Rad) as instructed by the manufacturer. A typical 20 µL ddRT‐PCR reaction contains 6 μL of diluted RNA sample, 5 μL of Supermix, 400 U of reverse transcriptase, 15 mmol/L dithiothreitol, and five primer‐probe sets at the optimized final concentrations (Set 1 or 2:900 nmol/L of primers and 250 nmol/L of probe; Set 3 or 4:360 nmol/L of primers and 100 nmol/L of probe; Set 5:900 nmol/L of primers, 250 nmol/L of Vic‐specific probe, and 100 nmol/L of Yama‐specific probe). The reaction mixture and 70 μL of droplet generation oil for probes (Bio‐Rad) were transferred to a droplet generation cartridge (DG8™, Bio‐Rad), and ddRT‐PCR droplets were generated by a droplet generator (QX200™ system, Bio‐Rad). Emulsified reactions were then transferred to a 96‐well PCR plate for ddRT‐PCR. The reactions were performed on a thermal cycler (T100™, Bio‐Rad) with the following conditions: reverse transcription at 50°C for 60 minutes, inactivation of reverse transcriptase, and activation of DNA polymerase at 95°C for 10 minutes, 40 cycles of PCR amplification (denaturation at 95°C for 30 seconds; annealing/extension at 60°C for 60 secondss), and PCR inactivation at 98°C for 10 minutes. Completed ddRT‐PCR reactions were kept temporarily at 4°C, and reaction signals were captured by a droplet reader (QX200™ system, Bio‐Rad). All reactions were required to yield a minimum of 10 000 droplets per reaction before downstream analyses. Data generated from the droplet reader were analyzed by a program designed for this platform (QuantaSoftTM Pro, version 1.0 BioRad). The gating strategy was set and optimized manually at the beginning of this study. The optimized gating strategy was used in all subsequent experiments. No‐template control reaction was included in each run.

### 2.4 Evaluation of six‐plex ddRT‐PCR

To test cross‐reactivity, six‐plex ddRT‐PCR were set‐up under different conditions: single and double positive using viral RNAs from virus stocks. The dynamic range of the assay was established using ten‐fold dilutions of viral RNAs from virus stocks. Each dilution was run in replicates on three different days, in which triplicates were performed on day 1 to show intra‐assay variability with a total of five replicates to show the inter‐assay variability.

Based on the dynamic range results, limit of quantification (LoQ) was set to be the lowest concentration of each targets that could be quantified with CV ≤ 25%. Sixteen replicates were run over three different days for the determination of LoQ of each target (triplicates on day 1, five replicates on day 2, and eight replicates on day 3). The limit of blank (LoB) was set to be the 95th percentile of positive droplets in reactions using the ten negative control samples, the samples were analyzed in duplicate to get twenty sets of data in total.

To compare the performance of this assay between single and double positive reactions, mixed samples were prepared using viral RNAs from two different virus stocks in various ratios. For one target, copies per reaction were very low (<99) or low (99‐250), whereas for the other target, copies per reaction were high (3000‐13 000) or very high (13 000‐70 000). The error was calculated by subtracting the quantitative data for a double positive reaction from that for a single positive reaction. The mean percentage error was calculated using the data in three independent runs.

### 2.5 Clinical specimens

Archived RNA samples extracted from human nasopharyngeal swabs (N = 55; sampling period: January and May of 2016) were tested. These samples were previously typed and classified by qRT‐PCR assays using protocols as previously described.^21^, ^22^, ^23^, ^24^ Briefly, viral RNA was extracted using QIAamp viral RNA mini kit (Qiagen) according to the manufacturer's instruction. qRT‐PCR was performed using Qiagen One‐Step RT‐PCR kit (Qiagen) and was conducted by thermal cycler (ViiA7 Real‐time PCR system, Thermo Fisher). RNA samples positive for H1pdm09 (N = 11), H3 (N = 12), Vic (N = 12), or Yam (N = 10), together with negative control samples (N = 10), were tested by the six‐plex ddRT‐PCR assay in a blinded format. Ct values for these samples in the above qRT‐PCR assays and the data generated from the ddRT‐PCR were analyzed in Prism8.

## 3 RESULTS

### 3.1 In silico analyses of primer and probe sequences

**FIGURE 1:**
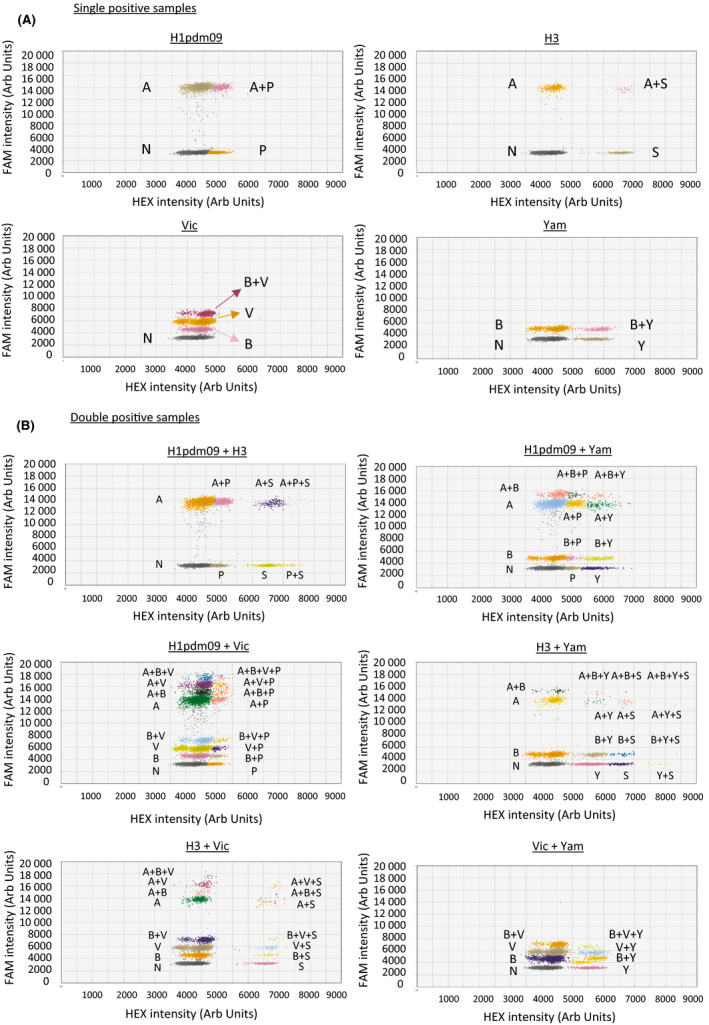
Cluster classification of (A) single positive and (B) double positive ddRT‐PCRs in 2D plots of droplet fluorescence, where the *x*‐axis and *y*‐axis are the fluorescence amplitude at channels HEX and FAM, respectively. The graph titles showed the type(s) of virus RNA added in the reaction

In this study, we developed a multiplex assay for detecting seasonal influenza viruses according to their respective types and subtypes/lineages. Probes targeting influenza A M gene, influenza B M gene, or influenza B Victoria HA gene were labeled with a FAM reporter, whereas probes targeting H1pdm09 HA gene, seasonal H3 HA gene, or influenza B Yamagata HA gene were labeled with a HEX reporter (Table 1). These primer and probe sequences were highly specific to contemporary influenza virus sequences (2009‐2019) and >97% of the studied influenza sequences should react with our sequence designs based on the criteria used in our study (see Materials and Methods). As the commercial ddRT‐PCR reader has only two channels for signal detection, we adopted the amplitude multiplexing technique to detect our targets.^15^ The primer‐probe sets were pre‐optimized to different concentrations so that the detected signals would form distinct clusters in a 2‐dimensional amplitude multiplexing plot (Figure 1).

### 3.2 Performance of the six‐plex assay for influenza virus detection

We first conducted the assay using viral RNA extracted from virus cultures. The extracted RNA from a particular strain was tested either alone or in mixture with another viral RNA in different combinations in order to make the studied reactions become single or double positive. As shown in Figure 1A, reactions containing viral RNA from different viruses yielded distinct signal cluster patterns. In double positive reactions, no cross‐reactivity was observed in all kinds of combinations (Figure 1B). It should be noted that we intended to use highly diluted RNA samples to achieve ≤ 1 copy per droplet for ddRT‐PCR. The numbers of double positive droplets were therefore expected to be low. If more concentrated RNA mixtures were used in this assay, the number of clusters would have increased (Figure S1).

For samples containing H1pdm09 and H3, no positive clusters were found in the FAM channel with fluorescence intensity under 12 000. In contrast, samples containing Vic and Yam had no positive clusters with FAM intensity of more than 8000. For double positive samples with the presence of both H1pdm09 and Vic, no positive cluster was found with HEX intensity of more than 5500. On the other hand, the sample having both H3 and Vic RNA had no positive cluster between 5000 and 5500 arbitrary units in the HEX channel. The sample containing the H1pdm09 and Yam had no positive cluster higher than 7000 arbitrary units in the HEX channel, but there was a positive cluster centered at 5000 arbitrary units in the HEX channel (Figure 1B). The sample with H3 and Yam RNAs had positive clusters from 5500 to 7000 arbitrary units in the HEX channel. The same interpretation rules indicated above were applied to reactions having higher concentrations of target samples (Figure S1B).

**FIGURE 2:**
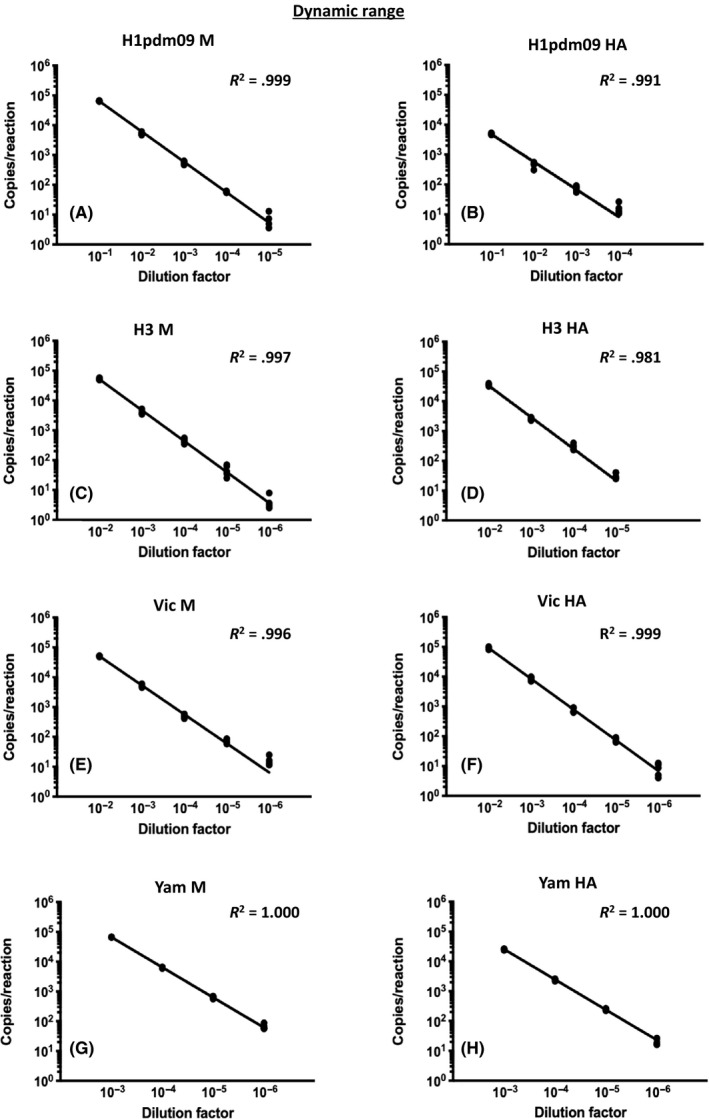
Dynamic range determined for four virus stocks. Data for a specific target are shown as indicated. Five replicates from three runs were performed for each dilution factor

We determined the dynamic range of the assay by using 10‐fold serially diluted RNA samples. The assay had a dynamic range of at least four orders of magnitude (Figure 2), which is typical for digital PCR assays with ~10 000 droplet/reaction.^25^ Results from this range of dilutions showed a linear relationship (*R* ^2^ ≥ 0.981). We also conducted multiple replicates to determine the intra‐assay variability (N = 3) and inter‐assay variability (N = 5). All tests had a CV value of less than 25%, with more dilute RNA samples tending to have a higher CV value as expected (Table 2). We further conducted tests on sixteen replicates to determine the limit of quantification (LoQ) of the assay (Table 3). The CV values of LoQ of this assay were all less than 25%, meeting the recommended standard for microbial detection.^26^ The LoQ values of this assay (copies/reaction) for our targets were as follows: H1pdm09 M: 29; H1pdm09 HA: 16; H3 M:23; H3 HA: 22; Vic M: 15; Vic HA: 20; and Yam M: 45 and Yam HA: 20.

**TABLE 2:** Intra‐ & inter‐assay reproducibility

	H1pdm09 M		H1pdm09 HA		H3 M		H3 HA		Vic M		Vic HA		Yam M		Yam HA	
	Intra‐assay															
Copies per reaction	Mean^a^	CV%	Mean^a^	CV%	Mean^a^	CV%	Mean^a^	CV%	Mean^a^	CV%	Mean^a^	CV%	Mean^a^	CV%	Mean^a^	CV%
×10^4^	6.43	1.2	N/A	N/A	5.15	5.6	3.76	6.0	5.04	3.6	8.65	7.9	6.66	0.3	2.60	1.0
×10^3^	5.62	7.6	4.97	7.0	3.92	14.7	2.51	8.0	5.17	14.2	8.32	17.9	6.49	1.0	2.51	1.1
×10^2^	5.77	9.8	5.11	7.8	4.53	23.7	3.37	15.5	4.87	9.0	7.45	18.2	6.40	1.0	2.47	6.1
×10^1^	5.69	3.0	8.35	9.2	3.41	24.0	3.21	22.3	7.63	20.3	8.15	12.1	6.06	9.5	2.02	24.0
	Inter‐assay															
Copies per reaction	Mean^b^	CV%	Mean^b^	CV%	Mean^b^	CV%	Mean^b^	CV%	Mean^b^	CV%	Mean^b^	CV%	Mean^b^	CV%	Mean^b^	CV%
×10^4^	6.55	2.2	N/A	N/A	5.35	8.2	3.53	8.1	5.14	1.7	9.50	8.4	6.67	0.3	2.53	5.6
×10^3^	5.04	9.9	4.86	3.6	4.31	18.3	2.58	11.2	5.44	5.7	8.94	7.2	6.37	6.1	2.43	9.2
×10^2^	5.57	14.4	4.29	25.3	4.57	10.6	2.74	20.2	4.97	17.5	7.60	18.8	6.33	11.0	2.44	7.9
×10^1^	5.83	5.5	6.76	21.5	4.73	24.2	3.25	23.9	6.88	9.9	7.01	14.4	6.81	24.6	2.11	23.4

^a^ No. of copies per reaction in 3 tests on same day.

^b^ No. of copies per reaction in 5 replicates on three different days.

**TABLE 3:** Limit of quantification

	Target							
	H1pdm09 M	H1pdm09 HA	H3 M	H3 HA	Vic M	Vic HA	Yam M	Yam HA
Mean^a^	29.3	15.8	22.7	22.3	14.6	19.5	45.2	19.5
Std. Deviation	7.0	3.4	5.2	5.5	3.3	4.4	7.7	4.8
Coefficient of variation	24.0%	21.4%	23.0%	24.5%	22.7%	22.5%	17.1%	24.8%

^a^ Average of sixteen replicates, in copies per reaction

In order to confirm that signals from one virus do not significantly interfere with those from another virus, we mixed two different viral RNA samples in various ratios and tested these mixtures using the ddRT‐PCR assay. The quantitative results were compared with those deduced from control reactions with RNA from a single virus. As shown in Table 4, the quantitative data of one target generated from mixed RNA samples were in concordance with the expected values (mean absolute percentage error ranged from 2.3% to 20.9%). These results indicated that this assay is suitable for identifying samples with dual influenza virus infections.

**TABLE 4:** Quantitative precision in tests with mixed samples

Copy number^a^				Mean absolute percentage error^b^					
H1pdm09	H3	Vic	Yam	FluA M	H1pdm09HA	H3 HA	FluB M	Vic HA	Yam HA
VH	VL			3.6%	2.3%	10.9%			
VH		L		10.0%	10.3%		15.0%	9.9%	
VH			VL	9.0%	10.2%		7.3%		13.1%
	H	L		6.8%		12.4%	17.8%	17.6%	
	H		VL	5.5%		8.5%	19.0%		9.7%
		VH	VL				5.1%	7.9%	11.7%
L	H			11.7%	14.6%	12.8%			
L		VH		18.7%	4.3%		4.2%	3.3%	
L			H	5.4%	12.2%		20.9%		13.9%
	VL	VH		8.6%		12.8%	11.6%	9.0%	
	VL		H	12.7%		13.2%	14.5%		9.1%
		L	H				18.1%	18.5%	12.7%

^a^ Copy number: less than 99 (very low, VL), 99 to 250 (low, L), 3000‐13000 (high, H), and 13000‐70000 (very high, VH).

^b^ The error was calculated by subtracting the copy number of a target in a double positive reaction from the copy number of the same target in a single positive reaction. Data are presented as mean absolute percentages of three runs.

Using RNA samples extracted from ten irrelevant respiratory clinical specimens as negative controls, we determined the number of false positive droplets in negative reactions (ie, limit of blank, LoB). The 95th percentile of positive droplets for FAM and HEX signals in a negative reaction (N = 20) were found to be 8 and 6, respectively. Thus, experimental reactions with numbers of positive droplets of less than 8 in FAM and 6 in HEX were considered as negative. Reactions with number of positive droplets above LoB, but below LoQ, are classified as “positive but not quantifiable.”

### 3.3 Evaluation of ddRT‐PCR assay using clinical specimens

**FIGURE 3:**
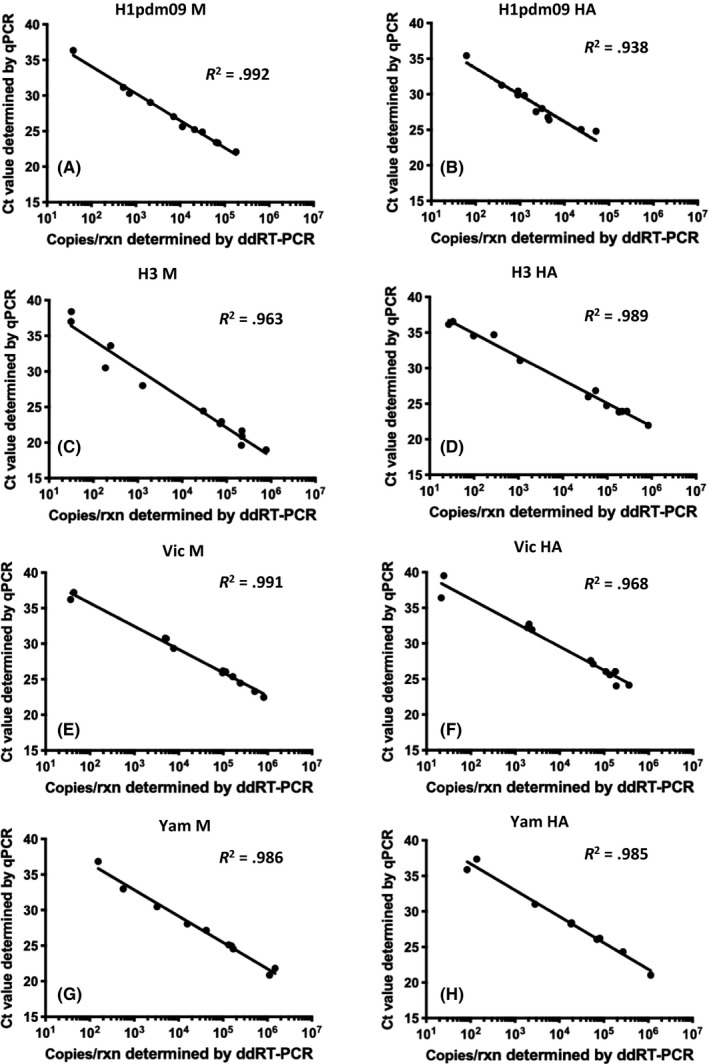
Linear regression between Ct values from qRT‐PCR and copies per reaction from ddRT‐PCR. Clinical samples positive for H1pdm09 (A and B), H3 (C and D), Vic (E and F), or Yam (G and H) were testeds

To evaluate the performance of this assay for clinical diagnosis, we tested 45 retrospective influenza‐positive and 10 control RNA samples extracted from nasopharyngeal swabs. These RNA samples were previously tested by influenza typing and subtyping using qRT‐PCR (See Methods and Materials), and double influenza infection was not detected in these samples. All typing and subtyping/lineage differentiation results generated from the ddRT‐PCR assay agreed with those deduced from the qRT‐PCR assays. We further compared results generated by the qRT‐PCR assay (Ct values) with those generated by ddRT‐PCR assays (copies per reaction). These two sets of data were highly correlated (Figure 3, *R* ^2^ > 0.938 for all targets). Overall, our results show that the ddRT‐PCR assay is a robust test for simultaneous typing, subtyping, and lineage determination of human influenza types A and B viruses.

## 4 DISCUSSION

Results from recent clinical studies indicate that influenza virus load can be a marker for disease severity.^9^ Influenza A and B viruses are often detected and quantified by qRT‐PCR in modern clinical settings.^7^ However, while this diagnostic approach is highly robust, qRT‐PCR is sensitive to inhibitors in clinical samples and absolute quantification of its target(s) is highly reliant on accuracy of the standard curve(s), resulting in significant inter‐laboratory variations.^27^ Hence, there is need to develop an accurate molecular test that does not require a standard curve for quantification.

In this study, we have developed a novel multiplex ddRT‐PCR for typing, subtyping, and lineage differentiation of human influenza viruses in a single reaction. As the QX200™ reader used in this study can recognize only two fluorophores simultaneously,^15^ we adopted an amplitude multiplexing approach to allow detection of six different targets in one reaction. The double‐quenched probe can provide flexibility on the probe length and reduce the background of detection.^28^ The six‐plex ddRT‐PCR, compared with the monoplex qRT‐PCR, can reduce reagent cost, handling time, and manpower and allow sample conservation.^15^, ^16^ Our results show that our targets positive signals display distinct patterns in a 2D analysis system (Figure 1). One should note that the complexity of clusters mainly depends on the concentration of targets in double positive reactions (Figure 1B and Figure S1B). Previous works from others showed that ddRT‐PCR technology can detect up to sixteen clusters with a maximum of four different targets in a single reaction.^15^ Thus, to the best of our knowledge, our ddRT‐PCR study is the first that shows formation of distinct clusters with the capacity to detect up to six targets in a typical positive reaction (Figure 1B), with a broad range of linearity (Figure 2) and good reproducibility within and between runs (Table 2). Further minor modifications of the primer‐probe sets or primer/probe ratio in future might help to enhance the detection limits of this assay.

The primer‐probe sets used in this study are designed to detect contemporary influenza A and B viruses. The influenza A M gene primer‐probe set targets a highly conserved region of M gene. This allows the assay to react with non‐human influenza A viruses, such as those from swine (data not shown). For samples that are strongly positive for M but negative for HA, further investigations such as sequencing are needed to rule out the possibility of zoonotic influenza infections.

Human cases caused by dual influenza virus infections are not unusual,^29^, ^30^, ^31^ but they are rarely detected in routine clinical settings by multiplex qRT‐PCR which suffers from competitive inhibition.^32^ This effect is more pronounced when there is a big difference between target concentrations,^33^ leading to a false negative result for the target at a lower concentration. By contrast, RNA studied in ddRT‐PCR assays was partitioned into >10 000 droplets, allowing a target at a low concentration to be amplified in a less competitive environment. Indeed, our results show that this assay can easily detect multiple targets at a disproportionate ratio (Table 4). We therefore believe that our ddRT‐PCR assay can avoid the above competitive inhibition issue and that this assay would be more useful in identifying cases with dual influenza virus infections. Recently, new ddPCR systems that can simultaneously detect four colors in a single reaction have become commercially available.^34^ This might create new opportunities to increase the number of targets per test, thereby allowing detection of pathogens in the same reaction.

The ddPCR system used here consists of three different modules: droplet preparation, thermal cycling, and droplet reading. This is a highly flexible platform which allows both low (N = 1) and high‐throughput analyses (N = 96). For a reaction strip of 8 reactions, the set‐up time is about 15‐20 minutes followed by 3 hours for RT‐PCR and 15 minutes to analysis results. The whole process takes approximately 4 hours for a reaction strip and 6 hours for a 96‐well plate. One should pay attention to the total droplet count for each reaction. For reactions with total droplet counts of less than 10 000, results cannot fulfill the assumption of Poisson statistics and therefore might be unreliable. Proper training can help to avoid these undesirable outcomes. In addition, further work is needed to determine whether this multiplex assay can be transferred to other ddPCR platforms. It is possible that additional optimization of the gating strategy for positive signals would be needed when the assay is conducted on other platforms.

Overall, this work shows our multiplex ddRT‐PCR assay is reliable and sensitive, allowing detection of six different targets simultaneously. It provides a useful tool for the study of human influenza viruses in clinical diagnosis and research laboratories.

## AUTHOR CONTRIBUTIONS

**Nathaniel K. C. Leong:** Conceptualization (equal); data curation (equal); formal analysis (equal); methodology (equal); writing‐original draft (equal). **Daniel K. W. Chu:** Data curation (equal); investigation (equal); resources (equal). **Julie T. S. Chu:** Formal analysis (equal); investigation (equal). **Yat H. Tam:** Methodology (equal); resources (equal). **Dennis K. M. Ip:** Investigation (equal); methodology (equal); resources (equal). **Benjamin J. Cowling:** Funding acquisition (supporting); investigation (equal); RESOURCES (equal). **Leo L. M. Poon:** Conceptualization (equal); formal analysis (equal); funding acquisition (equal); investigation (equal); project administration (equal); supervision (equal); writing‐original draft (equal); writing‐review and editing (equal).

## Supporting information

Figure S1
