# Supplementary material for: A six‐plex droplet digital RT‐PCR assay for seasonal influenza virus typing, subtyping, and lineage determination
Source: Influenza Other Respir Viruses. 2020 Jun 10;14(6):720–9. doi: 10.1111/irv.12769 (PMC7578307; doi:10.1111/irv.12769)
Supplement: Supplementary file 1 — Figure S1 [file IRV-14-720-s001.pptx]

## Slide 1
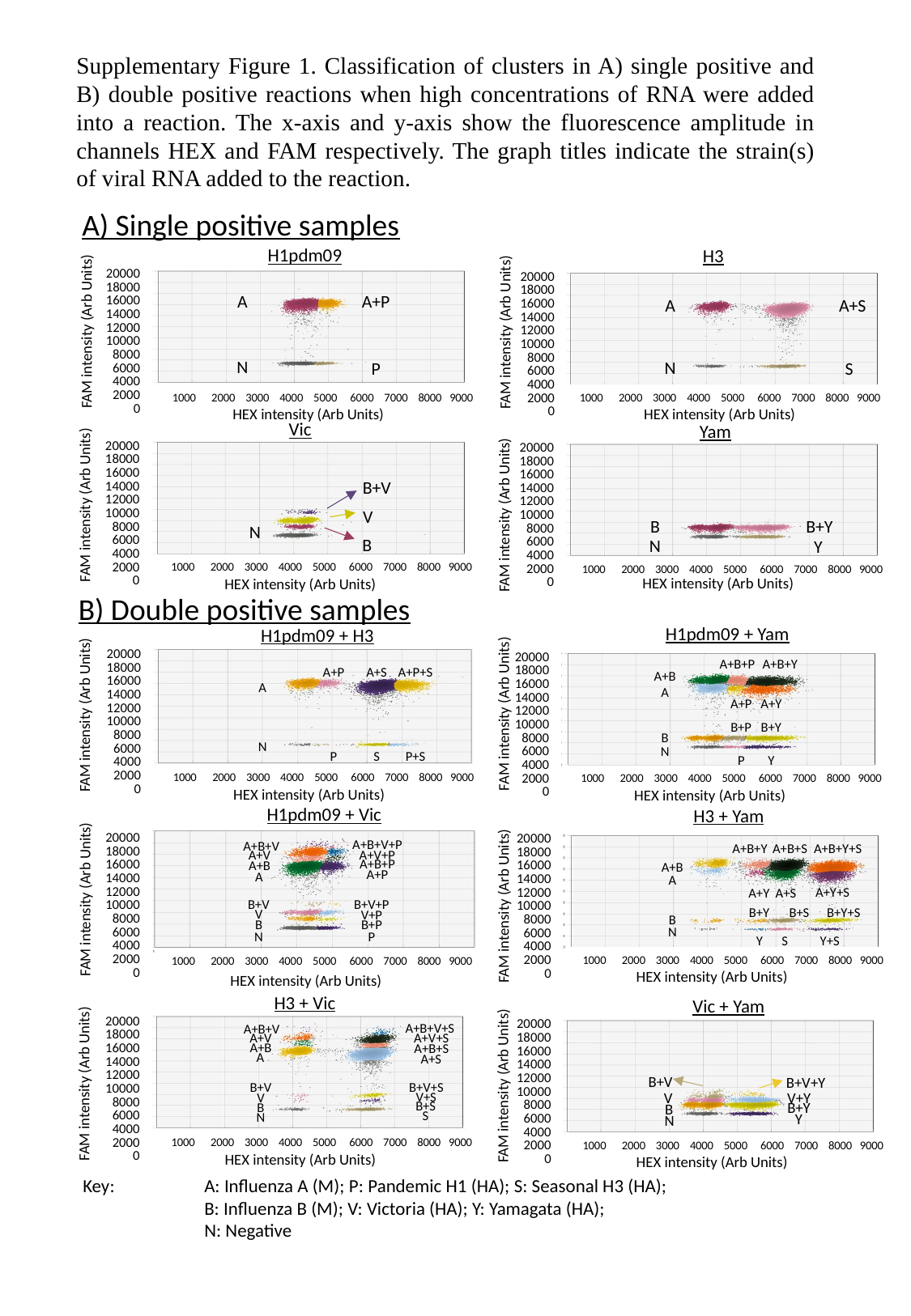

Supplementary Figure 1. Classification of clusters in A) single positive and B) double positive reactions when high concentrations of RNA were added into a reaction. The x-axis and y-axis show the fluorescence amplitude in channels HEX and FAM respectively. The graph titles indicate the strain(s) of viral RNA added to the reaction.
A) Single positive samples
H3
20000
18000
16000
14000
12000
10000
8000
6000
4000
2000
0
A
A+S
FAM intensity (Arb Units)
N
S
 1000 2000 3000 4000 5000 6000 7000 8000 9000
HEX intensity (Arb Units)
H1pdm09
20000
18000
16000
14000
12000
10000
8000
6000
4000
2000
0
A
A+P
N
P
 1000 2000 3000 4000 5000 6000 7000 8000 9000
HEX intensity (Arb Units)
Vic
20000
18000
16000
14000
12000
10000
8000
6000
4000
2000
0
B+V
V
N
B
 1000 2000 3000 4000 5000 6000 7000 8000 9000
HEX intensity (Arb Units)
Yam
20000
18000
16000
14000
12000
10000
8000
6000
4000
2000
0
B
B+Y
N
Y
 1000 2000 3000 4000 5000 6000 7000 8000 9000
HEX intensity (Arb Units)
B) Double positive samples
H1pdm09 + Yam
20000
18000
16000
14000
12000
10000
8000
6000
4000
2000
0
A+B+P
A+B+Y
A+B
A
A+P
A+Y
B+P
B+Y
B
N
P
Y
 1000 2000 3000 4000 5000 6000 7000 8000 9000
HEX intensity (Arb Units)
H1pdm09 + H3
20000
18000
16000
14000
12000
10000
8000
6000
4000
2000
0
A+P
A+S
A+P+S
A
N
P
S
P+S
 1000 2000 3000 4000 5000 6000 7000 8000 9000
HEX intensity (Arb Units)
H1pdm09 + Vic
20000
18000
16000
14000
12000
10000
8000
6000
4000
2000
0
A+B+V+P
A+B+V
A+V+P
A+V
A+B+P
A+B
A+P
A
B+V
B+V+P
V
V+P
B+P
B
N
P
 1000 2000 3000 4000 5000 6000 7000 8000 9000
HEX intensity (Arb Units)
H3 + Yam
20000
18000
16000
14000
12000
10000
8000
6000
4000
2000
0
A+B+Y
A+B+S
A+B+Y+S
A+B
A
A+Y+S
A+S
A+Y
B+Y
B+S
B+Y+S
B
N
Y
S
Y+S
 1000 2000 3000 4000 5000 6000 7000 8000 9000
HEX intensity (Arb Units)
H3 + Vic
20000
18000
16000
14000
12000
10000
8000
6000
4000
2000
0
A+B+V
A+V
A+V+S
A+B
A+B+S
A
A+S
B+V
B+V+S
V+S
V
B+S
B
S
N
 1000 2000 3000 4000 5000 6000 7000 8000 9000
HEX intensity (Arb Units)
Vic + Yam
20000
18000
16000
14000
12000
10000
8000
6000
4000
2000
0
B+V
B+V+Y
V
V+Y
B+Y
B
Y
N
 1000 2000 3000 4000 5000 6000 7000 8000 9000
HEX intensity (Arb Units)
A+B+V+S
Key: 	A: Influenza A (M); P: Pandemic H1 (HA); S: Seasonal H3 (HA);
	B: Influenza B (M); V: Victoria (HA); Y: Yamagata (HA);
	N: Negative
FAM intensity (Arb Units)
FAM intensity (Arb Units)
FAM intensity (Arb Units)
FAM intensity (Arb Units)
FAM intensity (Arb Units)
FAM intensity (Arb Units)
FAM intensity (Arb Units)
FAM intensity (Arb Units)
FAM intensity (Arb Units)
